# Indole Acetic Acid: A Key Metabolite That Protects Marine *Sulfitobacter mediterraneus* Against Oxidative Stress

**DOI:** 10.3390/microorganisms13051014

**Published:** 2025-04-28

**Authors:** Yongliang Gan, Runlin Cai, Guanjing Cai, Jude Juventus Aweya, Jianmin Xie, Ziming Chen, Hui Wang

**Affiliations:** 1 Guangdong Provincial Key Laboratory of Marine Biotechnology and Biology Department, College of Science, Shantou University, Shantou 515063, China; 2 Fujian Provincial Key Laboratory of Food Microbiology and Enzyme Engineering, College of Ocean Food and Biological Engineering, Jimei University, Xiamen 361021, China

**Keywords:** bacterial–algal interaction, marine microbiome, oxidative stress, indole acetic acid, amino acid metabolism, *Sulfitobacter mediterraneus*

## Abstract

For marine bacteria, the phycosphere is attractive as a major source of labile nutrients, but it also presents challenges due to the accumulation of stressors, such as reactive oxygen species (ROS) from algal metabolisms. Therefore, successful colonization of bacteria in the phycosphere requires an efficient mechanism to fight against oxidative stress, which is still a missing piece in studying bacteria–algae interactions. Here, we demonstrate that a common metabolite, indole acetic acid (IAA), enables the Roseobacter clade *Sulfitobacter mediterraneus* SC1-11, an IAA-producer, to resist hydrogen peroxide (H_2_O_2_) stress and that IAA biosynthesis can be activated by low concentrations of H_2_O_2_. Proteomics and metabolomics analyses revealed that bacteria consume high amino acid levels when exposed to H_2_O_2_ stress, while exogenous supplementation with IAA effectively protects bacteria from ROS damage and alleviates amino acid starvation by upregulating several proteins responsible for replication, recombination, and repair, as well as two proteins involved in amino acid transport and metabolism. Furthermore, the supplementation of some amino acids, such as arginine, also showed a significant protective effect on bacteria under H_2_O_2_ stress. This study highlights an unprecedented role of IAA in regulating amino acid metabolisms for resisting oxidative stress, which may be a specific strategy for adapting to the phycosphere.

## 1. Introduction

Interactions among microorganisms greatly contribute to biochemical cycling in marine ecosystems. The interactions between phytoplankton and associated bacteria occur on algal cells’ surfaces in a diffusive boundary layer with accumulating chemical molecules, termed the phycosphere [1]. Metabolic exchanges, including growth substrates, essential vitamins [2,3], and growth-promoting hormones [4] in the phycosphere, can benefit both interaction pairs. However, the phycosphere also possesses a plethora of harmful byproducts (e.g., reactive oxygen species, ROS) generated by algal and other microbial metabolisms [5,6,7,8]. In addition, the individual phycosphere of phytoplankton is an open ecosystem with various challenging environmental stresses, such as allelopathy [9], ultraviolet (UV) light [10], photo-acclimation [11], and oxidative stress [12,13]. These stresses threaten bacteria and limit how they thrive in the phycosphere. Thus, our present studies aim to understand how symbiotic bacteria respond and adapt to such challenges in the phycosphere, leading to a deeper understanding of the recruitment, assembly, and succession of microbiomes in the phycospheres of phytoplankton [14,15].

ROS accumulation contributes to an imbalance of redox metabolism in organisms during stress stimulation, which is characterized by ROS exceeding the cell’s capacity to detoxify. Oxidative stress can lead to a wide range of damage to bacterial cells, affecting various layers—from individual biomolecules, such as nucleotides and amino acids, to larger macromolecules, such as DNA, RNA, and proteins, which involve multiple biomolecular processes [16]. The multiple detrimental effects of oxidative stress on bacterial cells highlight the importance of addressing this issue in various biological contexts. In response to ROS, bacteria employ various oxidant scavenging strategies, such as enzymes (e.g., catalase) and the production of antioxidants (e.g., glutathione, GSH) [16,17]. Generally, bacteria deploy two interconnected stress response pathways, i.e., the general stress response and the stringent response, to coordinate the stress response by inducing the expression of protective detoxification, repair, and metabolic programs [16].

In rhizosphere models, indole acetic acid (IAA), a hormone that promotes plant growth classified as an auxin, is a common metabolic product of both plants and associated bacteria [18]. Bacterial auxin can manipulate plant growth by providing bacteria with access to nutrients and modulating the interaction between bacteria and plant immune system, a ROS-dependent defense response, ultimately promoting bacterial colonization in plants [19,20]. For example, *Bacillus velezensis*, a beneficial bacterium that secretes auxin, triggers the plant immune response. This, in turn, stimulates the bacterium to produce more auxin, which promotes bacterial survival and efficient root colonization [19,20]. However, whether phycosphere bacteria also employ such a mechanism to overcome oxidative stress remains unclear.

Despite successful detection of IAA in various microalgal phyla, including 20 algal strains in the Haptophyta, Chlorophyta, and Streptophyta [21], the biosynthetic pathways for IAA in microalgae remain complex and incompletely understood. A comprehensive survey of 16 representative algal genomes revealed that most marine algae analyzed lack complete IAA-biosynthetic genes [22]. For example, among the three analyzed diatom strains, *Fragilariopsis cylindrus* does not possess any IAA-biosynthetic gene homologs, while the other two strains (*Pseudo-nitzschia multiseries* CLN-47 and *Phaeodactylum tricornutum*) only lack key genes encoding tryptophan aminotransferase (L-amino acid oxidase) and tryptophan decarboxylase. However, recent studies have provided compelling genetic evidence that some microalgae are capable of synthesizing IAA, such as *Chlamydomonas*, which produces it via oxidation of tryptophan by L-amino acid oxidase [23,24]. Therefore, it is plausible that marine algae produce IAA through alternative pathways distinct from those in terrestrial plants. In addition, algal-associated bacteria are a significant source of IAA in the phycospheres of marine algae [1]. Recently, many studies have highlighted the significance of IAA as a signaling molecule in the interactions between microalgae and bacteria. For instance, IAA has been identified as a key molecule mediating cross-kingdom communication between algae and bacteria in aquatic environments [22]. This auxin produced by microalgae facilitates mutualistic interaction with bacteria [23]. Conversely, the role of bacteria-produced IAA acting as a signaling molecule in promoting algal growth [4,25,26] and photosynthesis has been well documented in bacterial–algal interactions [27], photosynthesis [4], and overall algal fitness [28]. While IAA-producing bacteria can promote algal growth, they also lead to the decline of aging algal hosts [29], a process also mechanistically modulated by IAA. Moreover, in the marine pathogenic bacterium *Vibrio campbellii*, indole and its derivatives (e.g., IAA) can modulate bacterial motility and biofilm formation [30], indicating that IAA has great potential regulatory effects on bacteria. The bacterial genus *Sulfitobacter*, a ubiquitous Gram-negative bacterium, has been identified as a representative mutualistic partner of phytoplankton. The bacteria in this genus exhibit the potential for IAA biosynthesis and algal growth-promoting capacity under natural and laboratory conditions [4,31,32]. The species *Sulfitobacter mediterraneus* was first identified in samples of natural seawater collected from the Mediterranean Sea [33]. Our laboratory previously isolated numerous strains affiliated with this species from the phycosphere of the marine diatom *Skeletonema costa* [34]. This diatom is known to have a positive relationship with its associated bacteria [35], which are capable of IAA biosynthesis [36]. While the role of IAA in the bacterial–algal interactions has been well described [4,26], the role that IAA plays in the physiology of these bacteria under stress conditions is not clear. In this study, we selected *Sulfitobacter mediterraneus* SC1-11, a bacterium closely associated with the marine diatom *Skeletonema costa* [34], as our model organism. Our primary objective was to conduct a comprehensive investigation into its physiological and biochemical responses under oxidative stress conditions. Furthermore, we aimed to elucidate the role of IAA in bacterial antioxidant defense mechanisms and metabolic regulation.

## 2. Methods and Materials

### 2.1. Bacterial Strains, Plasmids, and Growth Conditions

To obtain robustly active bacterial cells, *Sulfitobacter mediterraneus* SC1-11, originally isolated from the phycosphere of *Skeletonema costatum* collected from surface seawater at the Pearl River estuary [34], was initially cultivated in marine broth medium 2216E (Difco, Shanghai, China). The cultivation conditions were set at 28 °C with shaking at 180 rpm. All subsequent assays were conducted using minimal salt medium (MSM; see Text S1) [37].

### 2.2. ROS Detection

ROS assays were performed using the Reactive Oxygen Species Assay Kit (S0033S, Beyotime, Nantong, China). SC1-11 cells grown to the exponential phase (OD_600_ of 0.6–0.8) were harvested, washed twice with PBS (137 mM NaCl, 2.7 mM KCl, 1.8 mM KH_2_PO_4_, 0.01 mM Na_2_HPO_4_) buffer, and incubated with fluorescent probe DCFH-DA (S0033S, Beyotime, China) in the dark for 20 min. After removing the excess fluorescent probe, the cells were immediately exposed to serial concentrations (0, 0.5, 1, 5, and 10 mM) of hydrogen peroxide (H_2_O_2_) and transferred to a 96-well plate. The ROS levels were determined using a multimode reader (Synergy HTX, BioTek, Winooski, VT, USA) with an excitation wavelength of 488 nm and an emission wavelength of 525 nm. Each treatment was performed in triplicate.

### 2.3. Detection of IAA Produced by S. mediterraneus SC1-11 Under Oxidative Stress

Aliquots (10 mL) of SC1-11 cells grown to the exponential phase were exposed to serial concentrations (0, 0.5, 1, 5, and 10 mM) of H_2_O_2_ for 2 h, harvested by centrifugation (4000 rpm, 4 °C), and washed with cold PBS. Cells were resuspended in 200 μL of 80% methanol and incubated at 80 °C for 10 min before being vortexed. The last two steps were repeated twice. Next, the samples were centrifuged at 12,000× *g* for 5 min, and the collected supernatants were filtered through a 0.22 µm membrane (Millipore, Billerica, MA, USA). Finally, the samples were analyzed using a liquid chromatograph mass spectrometer (HPLC–MS) system with an Agilent 1260 HPLC (Agilent Technologies, San Diego, CA, USA) and Bruker amaZon SL (Bruker Daltonik GmbH, Bremen, Germany) in the positive ion mode. The LC column was a Luna 5u C18(2) column (100 A, 150 × 4.60 mm). The mobile phase consisted of A (0.1% formic acid in water) and B (acetonitrile) at a 1 mL/min flow rate. The gradient elution started with 5% B, was increased linearly to 100% B over 22 min, and was held for 3 min. The gradient elution was further decreased linearly to 5% B over 5 min to complete the analysis. Each treatment was performed in triplicate.

### 2.4. RNA Extraction and RT-qPCR

To analyze the expression of IAA-biosynthetic genes of SC1-11 under oxidative stress, bacterial cells in the exponential phase (OD_600_ between 0.6 and 0.8) were exposed to different concentrations (0, 0.5, 1, 5, and 10 mM) of H_2_O_2_ for 2 h. Total RNA was extracted from samples with the OMEGA E.Z.N.A.^®^ Total RNA Kit I (OMEGA Bio-tek, Norcros, GA, USA). After removing residual DNA from the total RNA, reverse transcription was performed using StarScript II RT Mix (GenStar, Bejing, China). Finally, the transcript levels of IAA-biosynthetic genes were analyzed using RT-qPCR performed with 2 × RealStar Green Fast Mixture (Genstar, Beijing, China). The 16S rRNA gene was used as an internal standard, and the relative expression of genes was determined using the 2^−ΔΔCt^ method [38].

### 2.5. Protective Effect of IAA Supplementation on H_2_O_2_-Induced Stress in S. mediterraneus SC1-11

To investigate the protective effect of IAA supplementation on strain SC1-11 in response to H_2_O_2_ treatment, bacteria were grown in 10 mL of 2216E overnight (OD_600_ ≈ 0.8), and 1 mL of cell culture was harvested, washed twice, and resuspended in PBS buffer, supplemented with various concentrations of IAA (i.e., 0, 57, 570, 5700 nM). Next, the cells were diluted in PBS containing 5-, 25-, and 125-fold dilutions of IAA, respectively. Aliquots (5 µL) of dilutions were spotted on 2216E plates containing 2 mM H_2_O_2_ (final concentration) before being incubated in the dark at 28 °C for 3 d, then photographed at 12 and 24 h.

**Figure 1:**
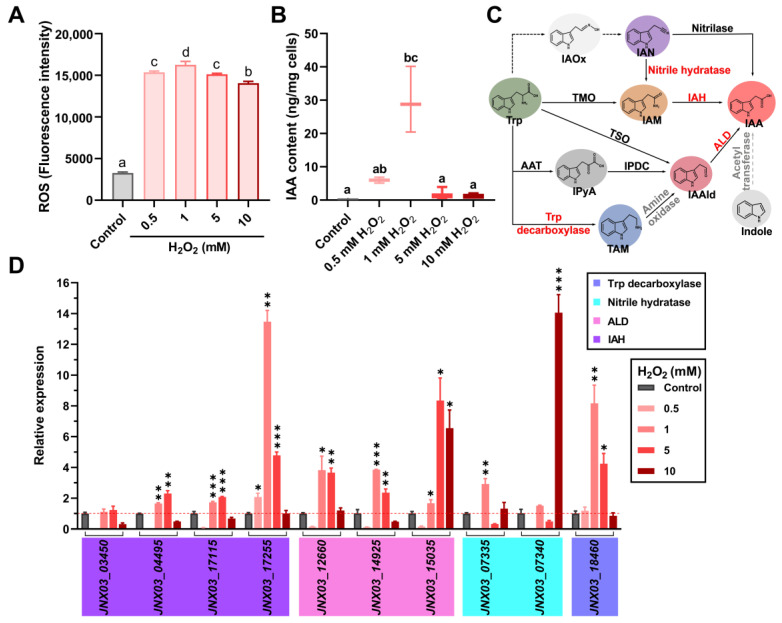
The responses of IAA biosynthesis to oxidative stress in *S. mediterraneus* SC1-11. ROS levels (**A**) and IAA production (**B**) of SC1-11 after exposure to varying concentrations of H_2_O_2_. Values are means ± SD, and lowercase letters indicate significant differences between treatments (by one-way analysis of variance (ANOVA)). (**C**) Overview of predicted IAA biosynthetic pathways in SC1-11. Enzymes indicated in red have been annotated in SC1-11, and other enzymes indicated in black or gray were reported or proposed in bacteria. Dashed lines indicate biochemical activities for which microbial enzymes have not been identified in bacteria. Abbreviations for metabolites and enzymes: Trp, tryptophan; IAOx, indole-3-acetaldoxime; IAN, indole-3-acetonitrile; IAM, indole-3-acetamide; IPyA, indole-3-pyruvate; IAAld, indole-3-acetaldehyde; TAM, tryptamine; AAT, amino acid aminotransferases; TMO, tryptophan 2-monoxygenase; IAH, indole-3-acetamide hydrolase; IPDC, indole-3-pyruvate dehydrogenase; ALD, indole-3-acetaldehyde dehydrogenase; TSO, tryptophan side chain oxidase. (**D**) RT-qPCR analysis of responses of IAA biosynthetic genes in SC1-11 under oxidative stress. Values are means ± SD, * *p* < 0.05, ** *p* < 0.01, *** *p* < 0.001 by unpaired *t*-test.

To examine the physiological and biochemical responses of SC1-11 to oxidative stress in the absence or presence of exogenous IAA, the ROS and malondialdehyde (MDA) contents of cells were assayed. To eliminate the influence of endogenous IAA, we carried out the subsequent assays with a treatment of 10 mM H_2_O_2_, as the production of IAA in cells was suppressed by higher concentrations of H_2_O_2_ (Figure 1B). Cells were grown to the exponential phase and then harvested, washed twice, and resuspended in PBS buffer supplemented with different concentrations (0–1.1 × 10^5^ nM) of IAA. After incubation for 30 min, 10 mM H_2_O_2_ was added, and cells were incubated in the dark at room temperature for 30 min before harvesting by centrifugation (12,000× *g*, 4 °C) and resuspension in PBS buffer. ROS levels were assayed as described above, and MDA levels were detected using the MDA assay kit (A003-1-2, Nanjing Jiancheng Bioengineering Institute, Nanjing, China). Each treatment was performed in triplicate.

### 2.6. Proteomics of S. mediterraneus SC1-11 Under Oxidative Stress

To explore the protective effect of IAA on SC1-11 against H_2_O_2_ at the proteome level, three groups were set up in triplicate: a control group (without any treatment), an H_2_O_2_ group (treatment with H_2_O_2_ only), and an IAA group (treatment with both H_2_O_2_ and IAA). Mid-exponential-phase SC1-11 cells were treated with 0.57 μM IAA for 30 min, followed by 10 mM H_2_O_2_ for 2 h. Cells were harvested (4000 rpm, 4 °C), washed twice with cold PBS, and resuspended in 500 µL lysis buffer (8 M urea, protease inhibitor (MBP002, Micron biolab, Atlanta, GA, USA)). Next, cells were broken by sonication on ice and centrifuged at 12,000× *g* to remove cellular debris. The total protein concentrations of the samples were determined using a BCA kit (BCAK-200, Micron Biolab, USA), according to the manufacturer’s instructions. Protein samples (100 µg) were treated with trypsin (V5280, Promega, Madison, WI, USA) after being diluted in seven volumes of water, and the peptides were further desalted and purified on C18 SPE cartridges (8B-S100-AAK, Phenomenex, Torrance, CA, USA) before being dried in a concentrator (Eppendorf, Hamburg, Germany). After solubilization in the solution (0.1% formic acid, acetonitrile/water (95/5, *v*/*v*)), samples were subjected to LC–MS/MS analysis on an Ultimate 3000 nanoRSLC (Thermo Fisher, Dreieich, Germany) system coupled to a Thermo Scientific Q Exactive HFX mass spectrometer, according to a previously described method [26].

The raw LC-MS/MS data were analyzed using Maxquant (v.1.5.2.8) with the following settings: Enzyme Name, Trypsin (Full); Precursor Mass Tolerance, 10 ppm; Fragment Mass Tolerance, 0.02 Da; Static Modification: Carbamidomethyl/+ 57.021 Da(C). A maximum of two missed cleavages was allowed. The maximum false discovery rate (FDR) for peptide and protein was specified as 0.01; the LFQ (Label-Free Quantitation) was enabled with an LFQ minimum ratio count of 1. Regression Settings: Regression Model, Non-linear Regression, Parameter Tuning, Coarse. The other settings were kept as default. Differentially expressed proteins (DEPs) were identified using the following screening criteria: Ratio ≤ 0.833 or ≥1.2, *p*-value < 0.05.

### 2.7. Quantification of S. mediterraneus SC1-11 Amino Acids Under Oxidative Stress

To analyze the changes in amino acid levels under oxidative stress, SC1-11 was precultured in 2216E to an OD_600_ between 0.6 and 0.8. Next, cells were transferred to 10 mL of MSM medium supplemented with different concentrations (0–1.1 × 10^5^ nM) of IAA and exposed to 10 mM H_2_O_2_ for 2 h in the dark. The protocol for amino acid extraction, separation, and detection was adapted from a previous study [39]. Briefly, the harvested bacterial pellets were resuspended in 200 µL of 80% ethanol at 80 °C. Samples were incubated at 80 °C for 10 min and then vortexed. These two steps were repeated twice. Samples were centrifuged at 12,000× *g* for 5 min, and the supernatants were collected before being filtered through a 0.22 µm membrane (Millipore). One microliter of sample was injected onto an HPLC–MS/MS system (Thermo TSQ-Endura, Thermo Fisher) in the positive ion mode. The LC column was a Hypersil GOLD^TM^ column (50 × 2.1 mm, 1.9 μm) maintained at 30 °C. The mobile phase consisted of A (methanol) and B (0.1% formic acid). Separation was achieved at a flow rate of 0.16 mL/min using the following gradient: 0–5 min at 90% B, followed by a 0.5 min ramping to 20% B, and then held constant for 4.5 min before returning to the initial condition (90% B). The total run time was 18 min. Each treatment was performed in triplicate.

### 2.8. Protective Effect of Amino Acid Supplementations on H_2_O_2_-Induced Effects in S. mediterraneus SC1-11

Mid-exponential-phase SC1-11 cultures were used for experiments to determine the protective effect of amino acids. The cultured cells were spun down, washed twice, and resuspended in an equal volume of PBS buffer containing a mixture of 20 proteinogenic amino acids (0.2 mM each). Next, the cell suspension was incubated with 5 mM H_2_O_2_ in the dark for 30 min before diluting with PBS buffer. Aliquots (5 μL) of the suspension and the diluted samples were spotted on 2216E plates. As a control, samples were treated similarly, without amino acid supplementation.

To investigate the protective effect of individual amino acid supplementations against H_2_O_2_, mid-exponential-phase SC1-11 cultures were centrifuged, washed twice, and resuspended in PBS. The suspensions were supplemented with 5 mM H_2_O_2_ (final concentration) and 10 mM individual amino acids (final concentration). Subsequently, 5 µL of the suspension and the diluted samples were spotted on 2216E plates before being incubated in the dark at 28 °C.

To further confirm the protective role of arginine, mid-exponential-phase SC1-11 cultures were subjected to a series of experiments. Initially, the bacterial culture was spun down, washed twice, and diluted in 10 mL of MSM medium (OD_600_ = 0.2) containing various concentrations (0–2.5 mM) of H_2_O_2_ and/or 10 mM arginine. Bacterial density (OD_600_) was determined using a multimode reader at different time points over 36 h.

In the experiment combining arginine with IAA, different concentrations (0, 5, and 10 mM) of arginine and 2.9 μM IAA were supplemented. The protective experiment was initially conducted on 2216E plates after SC1-11 cells were exposed to a higher (10 mM) concentration of H_2_O_2_. Additionally, the protective effect of arginine with IAA on cells was also confirmed in an MSM medium containing 1.5 mM H_2_O_2_. Due to the intrinsic ability of bacteria to eliminate H_2_O_2_, 1 mM H_2_O_2_ was added after a 4 h incubation to maintain oxidative pressure.

### 2.9. Bioinformatics Analysis

The whole genome sequencing of *S. mediterraneus* SC1-11 (GCA_016801775.1) was performed by a commercial company (Nextomics Biosciences, Wuhan, China) using Nanopore PromethION. De novo assembly of the genome used the software Flye v2.6 [40]. The protein-coding genes were predicted and annotated by the NCBI Prokaryotic Genome Annotation Pipeline (PGAP) or Rapid Annotation using Subsystem Technology (RAST, https://rast.nmpdr.org/rast.cgi (accessed on 14 July 2022)). Functional genes and metabolic pathways related to IAA biosynthesis and arginine–spermidine metabolism were annotated using Eggnog-mapper [41], Kyoto Encyclopedia for Genes and Genomes (KEGG) [42], and NCBI nonredundant protein (NR) databases.

### 2.10. Statistical Analyses

Statistical analyses were conducted using GraphPad Prism (version 9). Statistical differences in experiments with two groups were explored via Student’s *t*-tests. For multiple comparisons, one-way analysis of variance (ANOVA) was performed. A difference was considered statistically significant when *p* < 0.05. Detailed statistical analysis descriptions for each are provided in figure legends.

## 3. Results

### 3.1. Oxidative Stress Stimulates the Biosynthesis of IAA in S. mediterraneus SC1-11

When *S. mediterraneus* SC1-11 was treated with different concentrations (0, 0.5, 1, 5, 10 mM) of H_2_O_2_ to determine the relationship between intracellular ROS levels and IAA production, ROS levels increased rapidly with the addition of H_2_O_2_, reaching a peak when 1 mM H_2_O_2_ was added, followed by a slight decline upon adding higher concentrations of H_2_O_2_ (Figure 1A). The attenuation in detected levels of ROS at higher concentrations of H_2_O_2_ could be due to the inhibition of intracellular esterase activity or the leakage of fluorescent probes caused by cell envelope damage, which results in a decrease in the fluorescent signals of ROS assays. Besides the changes in ROS levels, the addition of different concentrations of H_2_O_2_ (from 0 to 1 mM) also progressively increased IAA production (Figure 1B), with higher H_2_O_2_ concentrations having a significant negative effect on IAA production partly because of the inhibition of metabolic activity. These observations suggest that IAA biosynthesis in SC1-11 could be linked to its adaptive responses.

When the IAA biosynthetic pathways in the strain SC1-11 were analyzed based on genome analysis, several IAA-biosynthetic genes that encode at least two independent pathways were successfully annotated (Figure 1C). A putative tryptophan decarboxylase gene (*JNX03_18460*), which catalyzes the conversion of tryptophan into tryptamine (TAM), was found in strain SC1-11. Similarly, three genes (*JNX03_12660*, *JNX03_14925*, and *JNX03_15035*) that encode the downstream acetaldehyde dehydrogenase catalyzing the conversion of indole acetaldehyde (IAAId) into IAA were successfully annotated. Another pathway, which is composed of nitrile hydratase (encoded by *JNX03_07335* and *JNX03_07340*) and indole acetamide hydrolase (IAH, encoded by *JNX03_03450*, *JNX03_04495*, *JNX03_17115*, and *JNX03_17255*), was also annotated. Next, when the expression of these genes under various oxidant concentrations (i.e., 0 to 10 mM H_2_O_2_) was analyzed by RT-qPCR, most of the IAA biosynthetic genes were significantly upregulated when 1 or 5 mM H_2_O_2_ was added (Figure 1D). These results suggest that oxidative stress can induce bacterial IAA biosynthesis.

### 3.2. IAA Supplementation Protects S. mediterraneus SC1-11 from Oxidative Stress

**Figure 2:**
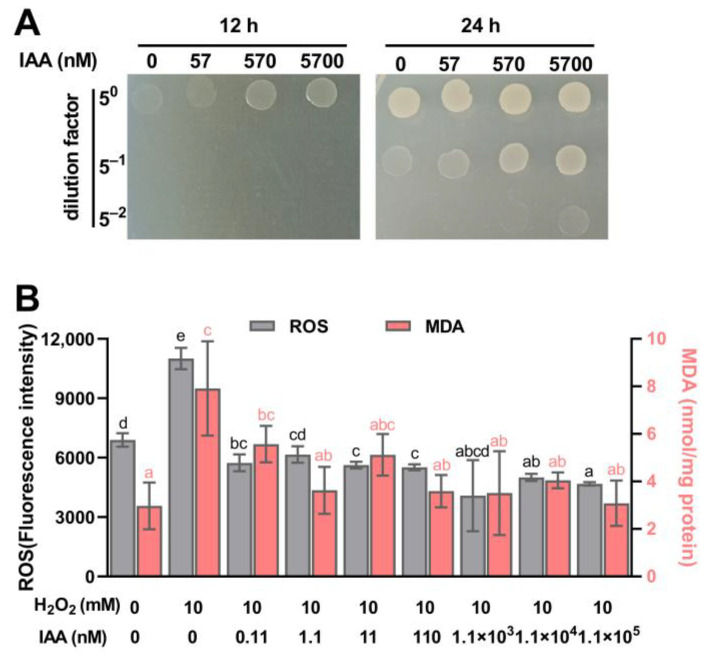
The protective role of IAA supplementation in *S. mediterraneus* SC1-11 against H_2_O_2_. (**A**) The growth of SC1-11 with the addition of IAA when exposed to 2 mM H_2_O_2_. (**B**) ROS and MDA levels of SC1-11 with incubation in varying concentrations of IAA and H_2_O_2_. Values are means ± SD, and lowercase letters indicate significant differences between treatments (ANOVA).

When SC1-11 was exposed to 2 mM H_2_O_2_, we observed that the addition of IAA (up to 570 nM) improved H_2_O_2_ tolerance in SC1-11 compared with lower concentrations of IAA (Figure 2A), suggesting that IAA has a dose-dependent protective role in SC1-11 against H_2_O_2_ stress. When ROS levels in SC1-11 were determined during H_2_O_2_ stress with IAA supplementation, we observed a sharp increase in ROS levels when SC1-11 was exposed to 10 mM H_2_O_2_, but ROS levels decreased to baseline levels after supplementing with various concentrations (0.11–1.1 × 10^5^ nM) of IAA (Figure 2B). Regarding levels of malondialdehyde (MDA), another physiological index and an end-product of lipid peroxidation, often used to assess bacterial health risk and oxidative damage [43], it was observed that significant increases in MDA levels were detected in the presence of H_2_O_2_ exposure only and/or the simultaneous addition of 0.11 nM IAA, compared to the control group. In contrast, no significant changes in MDA levels were noted when IAA concentrations added exceeded 1.1 nM. This further confirmed that IAA can protect SC1-11 from oxidative damage. It is noteworthy that the IAA concentrations added at the micromolar level, which are significantly lower than that of the oxidant (10 mM H_2_O_2_), could still create considerable ROS protection, suggesting that the protective effect of IAA against ROS may be attributed to the enhancement of cellular defense responses rather than the quenching of oxidants.

### 3.3. Oxidative Stress-Induced Proteomic and Metabolic Changes in SC1-11 Are Ameliorated by IAA Supplementation

**Figure 3:**
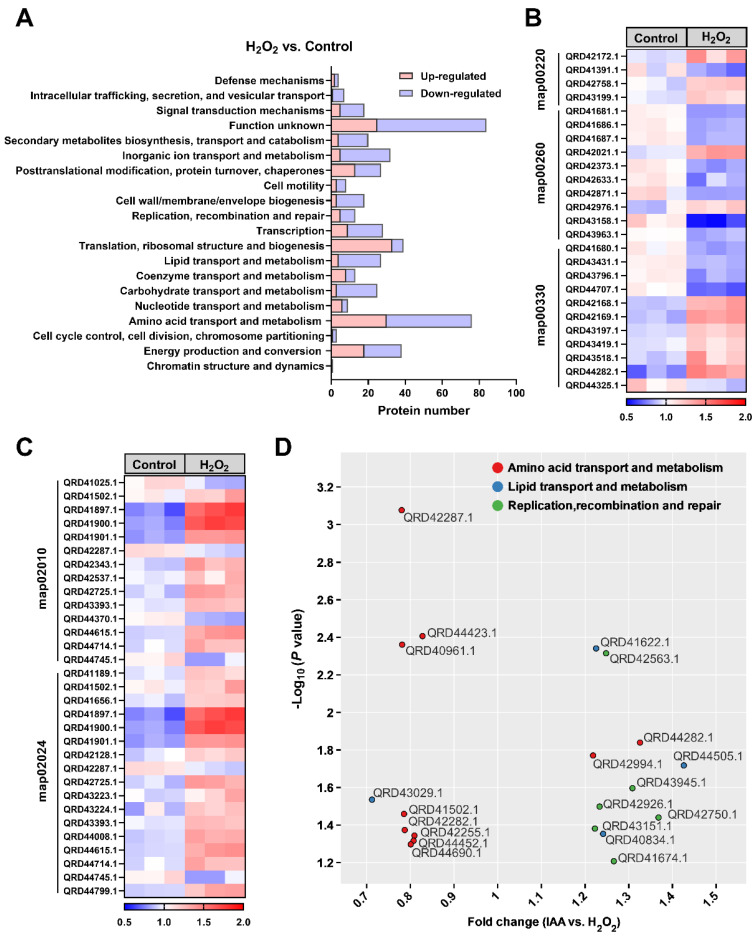
Proteomic responses of *S. mediterraneus* SC1-11 to 10 mM H_2_O_2_ before and after adding 0.57 μM IAA. (**A**) Differentially expressed proteins (DEPs) were categorized based on the Clusters of Orthologous Groups (COG) database. (**B**,**C**) Heatmaps of a subset of DEPs involved in amino acid transport and metabolism. (**D**) The DEPs enriched in amino acid transport and metabolism, lipid transport and metabolism, as well as replication, recombination and repair, are shown.

The effect of IAA on SC1-11 survival was examined during oxidative stress by determining changes in the proteome upon exposure to H_2_O_2_. Proteomic profiling revealed 2915 proteins, of which 498 (177 upregulated and 321 downregulated) significantly differed between the H_2_O_2_ treatment and the control (Supplementary Figure S1A). The differentially expressed proteins (DEPs) were classified into 20 functional categories according to the Cluster of Orthologous Groups of Proteins (COG) classification (Figure 3A). The top five COG categories were function unknown (16.9%), amino acid transport and metabolism (15.3%), translation, ribosomal structure and biogenesis (7.8%), energy production and conversion (7.6%), and inorganic ion transport and metabolism (6.4%). Interestingly, the upregulated proteins were also enriched in these functional categories, including translation, ribosomal structure and biogenesis (18.6%); amino acid transport and metabolism (16.9%); and energy production and conversion (10.2%), indicating fundamental physiological and functional changes in SC1-11 cells during oxidative stress responses. When the proteins related to amino acid transport and metabolism were sub-classified according to KEGG categories, the enriched categories included map00220 (Glycine, serine and threonine metabolism), map00260 (Glycine, serine and threonine metabolism), map00330 (Arginine and proline metabolism), map02010 (ABC transporters), and map02024 (Quorum sensing) (Figure 3B,C and Supplementary Figure S1). Unexpectedly, the pathways map00330 (Arginine and proline metabolism), map02010 (ABC transporters), and map02024 (Quorum sensing) were activated during oxidative stress, inconsistent with a previous finding in the Gram-positive bacterium *Bacillus valezensis* FZB42 [19].

The introduction of IAA into the culture medium significantly upregulated the expression of 36 proteins and downregulated 29 proteins, with proteins involved in replication, recombination and repair mainly significantly upregulated (Figure 3D and Supplementary Figure S1B), consistent with the protective role of IAA. Although there were changes in the expression of proteins associated with amino acid transport and metabolism, only two proteins, i.e., QRD42994.1, a putative glycine betaine/proline transport system ATP-binding protein, and QRD44282.1, a putative arginase family protein, were upregulated (Figure 3D and Supplementary Figure S1B), indicating that IAA could be beneficial in the reprogramming of partial amino acid metabolism in bacteria.

**Figure 4:**
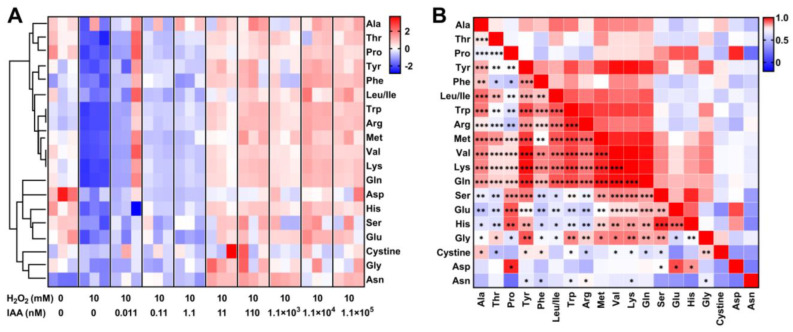
Changes in amino acid levels under oxidative stress without/with addition of varying concentrations of IAA. (**A**) Heatmap of normalized amino acid levels across different treatments. Abbreviations for amino acids: Ala, alanine; Asp, aspartic acid; Glu, glutamic acid; Phe, phenylalanine; Gly, glycine; His, histidine; Leu/Ile, leucine or isoleucine; Lys, lysine; Met, methionine; Asn, asparagine; Pro, proline; Gln, glutamine; Arg, arginine; Ser, serine; Thr, threonine; Val, valine; Trp, tryptophan; Tyr, tyrosine. (**B**) Heatmap of correlation between amino acids. Colors indicate the Pearson’s correlation value and significant correlations are marked with asterisks. * *p* < 0.05, ** *p* < 0.01, *** *p* < 0.001.

Next, we asked whether the intracellular amino acid levels of SC1-11 could be linked to its oxidant responses. When the changes in amino acid levels in SC1-11 under H_2_O_2_ stress were determined, 14 out of 19 (except for cysteine) amino acids and a dipeptide cystine, which is transformed from cysteine by cystine reductase, were significantly decreased (Figure 4A), suggesting that the specific metabolic changes in amino acid levels could play an important role against oxidative stress. The intracellular amino acid levels in SC1-11 increased gradually with IAA supplementation (0.011 to 1.1 × 10^5^ nM). Pearson’s correlation analysis revealed that 16 amino acids (except for asparagine and aspartic acid) had positive correlations (Pearson’s coefficient *r* > 0.5, Figure 4B), with 14 amino acids displaying stronger correlations (*r* > 0.75, *p* < 0.05), particularly tryptophan, arginine, lysine, and glutamine (*r* > 0.82, *p* < 0.01). In contrast, aspartic acid and asparagine correlated negatively (*r* = −0.13). These results suggest that in SC1-11, the depletion and metabolism of amino acids are strongly linked with bacterial anti-oxidative responses, and IAA may serve as a signal molecule that affects the metabolic allocation of amino acids.

### 3.4. Amino Acids Supplementation Protects S. mediterraneus SC1-11 Against Oxidative Stress

**Figure 5:**
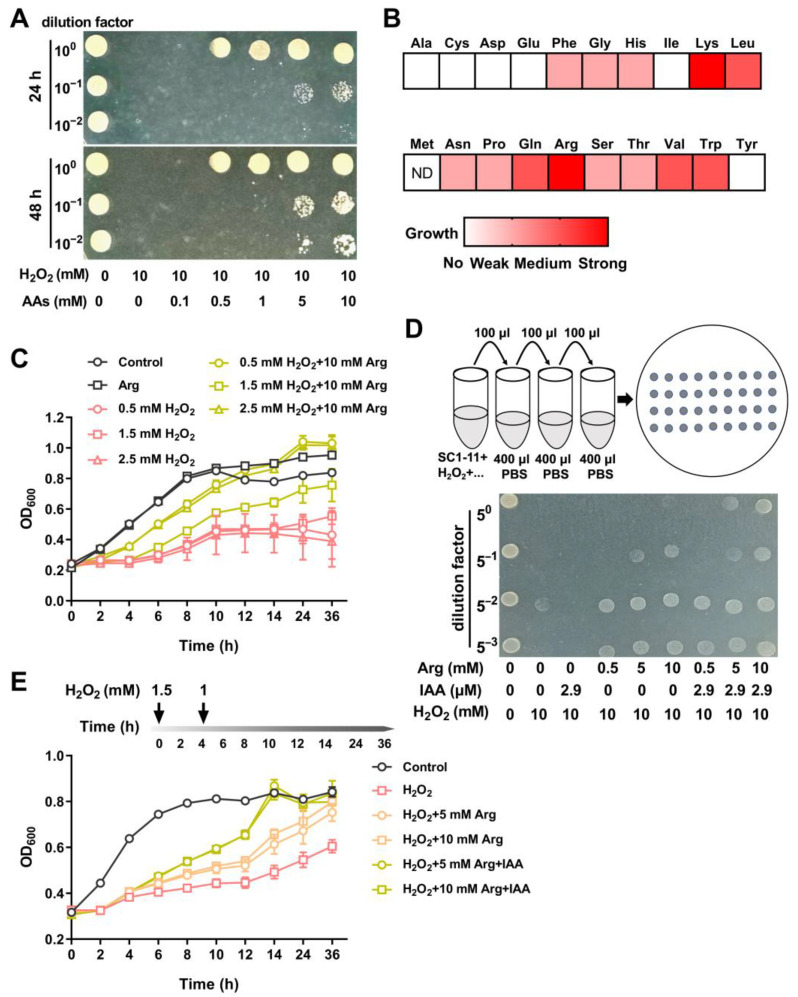
The protective role of amino acid supplementation in *S. mediterraneus* SC1-11 against H_2_O_2_. (**A**) The survival of SC1-11 with the addition of a mixture of 20 amino acids (AAs) after incubation in 10 mM H_2_O_2_. (**B**) Heatmap of bacterial growth incubated with individual amino acids when exposed to 10 mM H_2_O_2_. Colors indicate growth strength of SC1-11 (white indicates no growth). Abbreviations for amino acids are as described above; Cys indicates cysteine. (**C**,**E**) Growth curves of SC1-11 with the addition of arginine (**C**) or both arginine and IAA (**E**). (**D**) The survival of SC1-11 with the addition of arginine and IAA after incubation in 10 mM H_2_O_2_.

Bacterial survival analyses were conducted during exposure to oxidants (e.g., H_2_O_2_) with supplementation of individual proteinogenic amino acids to explore the role and physiological importance of amino acids in SC1-11 during oxidative stress. When the impact of a mixture of 20 amino acids (0.5 to 10 mM each) on bacterial survival under oxidative stress was determined, SC1-11 exhibited significant survival and growth in the presence of the amino acid mixture, suggesting the presence of at least partial amino acids conferred a strong tolerance to H_2_O_2_ in SC1-11 cells (Figure 5A). However, single amino acid supplementation resulted in variable bacterial survival. A heatmap of bacterial growth differences revealed 13 proteinogenic amino acids that could dramatically promote the growth of SC1-11 under H_2_O_2_ stress (Figure 5B and Supplementary Figure S2). Notably, lysine and arginine strongly protected SC1-11 against H_2_O_2_ stress. Moreover, when SC1-11 was cocultured with arginine under varying concentrations of H_2_O_2,_ the growth of SC1-11 cells was significantly enhanced (Figure 5C).

Given that individual IAA or amino acid supplementation could protect SC1-11 cells, especially arginine supplementation, and that arginine metabolism pathway COG categories were enriched in response to oxidative stress (Figure 5B and Supplementary Figure S1), we examined whether supplementation with both arginine and IAA would provide better protection against oxidative stress. Using growth experiments with plate and liquid systems, we observed that the combination of arginine and IAA had a better protective effect (Figure 5D,E). In the liquid system, the growth of SC1-11 cells was significantly promoted after the addition of arginine and IAA, peaking at 14 h, and with a maximal increase in optical density of 69.6% for 10 mM arginine plus IAA and 76.1% for 5 mM arginine plus IAA, compared to the H_2_O_2_-treated group (Figure 5E). Taken together, these results suggest that amino acid uptake and metabolism play a pivotal role in protecting strain SC1-11 against oxidants.

## 4. Discussion

Bacteria in the Roseobacter clade, such as *Sulfitobacter* spp., are known to engage in mutualistic relationships with marine microalgae [4,29,31], wherein the microalgae secrete various organic matters that meet the nutrient requirements of their symbiotic bacteria. In turn, the bacteria can transform tryptophan from algal exudates into IAA, an important plant growth-promoting hormone that is beneficial to the algal cells [4]. Here, we discovered that in addition to the host cells, IAA also benefits the diatom-associated bacteria. Our data revealed a close relationship between IAA production in strain SC1-11 and intracellular ROS levels, implying IAA’s potential in ROS protection. Bacterial growth media supplemented with IAA enhanced cell survival and resilience under oxidative stress, likely due to IAA’s ability to protect against ROS. This observation is consistent with a recent finding in a plant-beneficial *Bacillus valezensis* FZB42, which demonstrated the bacterium’s capacity to counteract the plant immune response that creates an ROS burst to eliminate microbial invaders [20], suggesting that the IAA-mediated ROS protection mechanism may be conserved in a wide range of bacteria. However, such mechanisms remain poorly known in the phycosphere system. To our knowledge, H_2_O_2_, which is commonly produced photochemically in surface waters at the oxic–anoxic interface and through biotic factors such as excretion from lactic acid bacteria [44] and metabolic byproducts from aging microalgae [8], is widely distributed in marine ecosystems. It is well known that IAA, a common molecule in phycosphere niches, has been found in a wide range of microalgae and their associated bacteria. The tryptophan-dependent pathways for IAA biosynthesis have been well documented in phycosphere bacteria [4,22,26] and some microalgae [22,23]. For example, *Chlamydomonas reinhardtii* can produce IAA from tryptophan using L-amino acid oxidase (LAO1) under nitrogen-limited conditions to inhibit algal cell multiplication and chlorophyll degradation [23]. Given this established mechanism in *Chlamydomonas*, it is plausible that other microalgae could also produce IAA, potentially through similar pathways. However, the specific pathways and enzymes involved in IAA production may vary among different microalgal species because most marine microalgae lack complete IAA-biosynthetic gene homologs [22].

The changes in protein and metabolite profiles suggest that oxidative stress may lead to significant depletion of amino acids, energy, and reducing coenzymes, likely due to a cascade of oxidative damage and subsequent defense mechanisms. Amino acids such as glycine, cysteine, and methionine serve as substrates for glutathione (GSH), an important antioxidant synthesized by bacteria in response to environmental stress [45,46]. Our data further revealed the contribution of several other amino acids, including tryptophan, to the antioxidant response of bacteria. Given that tryptophan functions both as a substrate for protein translation and as a primary precursor for IAA biosynthesis [4], this explains why both IAA and tryptophan supplementation protected bacteria from ROS toxicity in our study. Additionally, the shift in bacterial–algal interactions from mutualistic to pathogenic has been shown to be closely associated with the regulation of tryptophan metabolism [4,29]. Intriguingly, our proteomic and metabolic analyses identified two positively charged amino acids, lysine and arginine, which exhibited strong protective effects against oxidative stress. Indeed, several studies have demonstrated that lysine and arginine can facilitate antioxidant activity, likely through conversion into antioxidant polyamines such as cadaverine, putrescine, spermidine, and spermine, which play significant antioxidant roles in both prokaryotic and eukaryotic organisms [39,47]. We observed a unique phenomenon where supplementation of arginine together with IAA provided enhanced ROS protection for bacterial cells. We also found that IAA supplementation upregulated an arginase family protein that converts arginine to ornithine. This suggests that IAA levels in bacteria may be involved in modulating arginine degradation metabolism. Additionally, most amino acids can be converted to alpha-keto acids by aminotransferases that are abundant in microorganisms [48]. Given that various alpha-keto acids, including pyruvate, oxaloacetate, and alpha-ketoglutarate, have been shown to possess significant H_2_O_2_-scavenging activity [49], it is reasonable that these could be transformed from amino acids and play a crucial role in the detoxification of ROS within SC1-11 cells.

Gram-negative bacteria typically rely on ATP-binding cassette (ABC) transporters for the uptake of nutrients and signaling molecules [50]. A recent study proposed that the transporter IadK2 may contribute to IAA uptake in the rhizosphere bacterium *Variovorax*, which is a highly IAA-specific ABC transporter solute-binding protein (SBP) that works with ABC transporters in the membrane and mediates IAA uptake [51]. Given that *S. mediterraneus* possesses abundant ABC transporters, it is plausible that this species may employ a similar mechanism for IAA uptake as observed in *Variovorax*. However, the presence and specific function of an IAA-specific ABC transporter in *S. mediterraneus* has yet to be confirmed.

This study provides compelling evidence for a new role of IAA in protecting the Roseobacter clade bacterium *S. mediterraneus* SC1-11 against ROS. Our findings demonstrate that IAA functions as a regulatory factor, reprogramming amino acid metabolism and ROS defense mechanisms to mitigate ROS damage. However, future work is needed to further confirm the role of IAA by providing more genetic evidence and to identify IAA-binding proteins and regulatory proteins. Additionally, elucidating the detailed molecular mechanisms by which IAA modulates bacterial central metabolism and antioxidant responses will be crucial for a comprehensive understanding of this process.

## Data Availability

The complete genome data of *S. mediterraneus* SC1-11 have been deposited in the NCBI database and are available under accession number: ASM1680177v1. The mass spectrometry proteomics data have been deposited in the ProteomeXchange Consortium (https://proteomecentral.proteomexchange.org (accessed on 23 June 2024)) via the iProX partner repository with the dataset identifier PXD052982.

## Supplementary Materials

The following supporting information can be downloaded at: https://www.mdpi.com/article/10.3390/microorganisms13051014/s1, Text S1: Composition of minimal salt medium; Figure S1: Proteomic responses of *S. mediterraneus* SC1-11 under peroxide hydrogen stress before and after treatment with indole acetic acid (IAA); Figure S2: Supplementation of individual amino acids plays a protective role against oxidative stress in *S. mediterraneus* SC1-11; Table S1: Primers used for this study.

## References

[1] Seymour J.R., Amin S.A., Raina J.-B., Stocker R. (2017). Zooming in on the phycosphere: The ecological interface for phytoplankton–bacteria relationships. Nat. Microbiol..

[2] Cooper M.B., Kazamia E., Helliwell K.E., Kudahl U.J., Sayer A., Wheeler G.L., Smith A.G. (2019). Cross-exchange of b-vitamins underpins a mutualistic interaction between *Ostreococcus tauri* and *Dinoroseobacter shibae*. ISME J..

[3] Croft M.T., Lawrence A.D., Raux-Deery E., Warren M.J., Smith A.G. (2005). Algae acquire vitamin b12 through a symbiotic relationship with bacteria. Nature.

[4] Amin S.A., Hmelo L.R., Van Tol H.M., Durham B.P., Carlson L.T., Heal K.R., Morales R.L., Berthiaume C.T., Parker M.S., Djunaedi B. (2015). Interaction and signalling between a cosmopolitan phytoplankton and associated bacteria. Nature.

[5] Astafyeva Y., Gurschke M., Qi M., Bergmann L., Indenbirken D., De Grahl I., Katzowitsch E., Reumann S., Hanelt D., Alawi M. (2022). Microalgae and bacteria interaction—Evidence for division of *Diligence* in the alga microbiota. Microbiol. Spectr..

[6] Zhang S., Zheng W., Wang H. (2020). Physiological response and morphological changes of *Heterosigma akashiwo* to an algicidal compound prodigiosin. J. Hazard. Mater..

[7] Seyedsayamdost M.R., Case R.J., Kolter R., Clardy J. (2011). The Jekyll-and-Hyde chemistry of *Phaeobacter gallaeciensis*. Nat. Chem..

[8] Deng Y., Yu R., Grabe V., Sommermann T., Werner M., Vallet M., Zerfaß C., Werz O., Pohnert G. (2024). Bacteria modulate microalgal aging physiology through the induction of extracellular vesicle production to remove harmful metabolites. Nat. Microbiol..

[9] Sukenik A., Eshkol R., Livne A., Hadas O., Rom M., Tchernov D., Vardi A., Kaplan A. (2002). Inhibition of growth and photosynthesis of the dinoflagellate *Peridinium gatunense* by *Microcystis* sp. (Cyanobacteria): A novel allelopathic mechanism. Limnol. Oceanogr..

[10] Rijstenbil J.W. (2002). Assessment of oxidative stress in the planktonic diatom *Thalassiosira pseudonana* in response to UVA and UVB radiation. J. Plankton Res..

[11] Li Z., Wakao S., Fischer B.B., Niyogi K.K. (2009). Sensing and responding to excess light. Annu. Rev. Plant Biol..

[12] Van Creveld S.G., Rosenwasser S., Schatz D., Koren I., Vardi A. (2015). Early perturbation in mitochondria redox homeostasis in response to environmental stress predicts cell fate in diatoms. ISME J..

[13] Thamatrakoln K., Korenovska O., Niheu A.K., Bidle K.D. (2012). Whole-genome expression analysis reveals a role for death-related genes in stress acclimation of the diatom *Thalassiosira pseudonana*. Environ. Microbiol..

[14] Thukral M., Allen A.E., Petras D. (2023). Progress and challenges in exploring aquatic microbial communities using non-targeted metabolomics. ISME J..

[15] Bell W., Mitchell R. (1972). Chemotactic and growth responses of marine bacteria to algal extracellular products. Biol. Bull..

[16] Fasnacht M., Polacek N. (2021). Oxidative stress in bacteria and the central dogma of molecular biology. Front. Mol. Biosci..

[17] Ezraty B., Gennaris A., Barras F., Collet J.-F. (2017). Oxidative stress, protein damage and repair in bacteria. Nat. Rev. Microbiol..

[18] Kunkel B.N., Harper C.P. (2018). The roles of auxin during interactions between bacterial plant pathogens and their hosts. J. Exp. Bot..

[19] Mashiguchi K., Hisano H., Takeda-Kamiya N., Takebayashi Y., Ariizumi T., Gao Y., Ezura H., Sato K., Zhao Y., Hayashi K. (2019). *Agrobacterium tumefaciens* enhances biosynthesis of two distinct auxins in the formation of crown galls. Plant Cell Physiol..

[20] Tzipilevich E., Russ D., Dangl J.L., Benfey P.N. (2021). Plant immune system activation is necessary for efficient root colonization by auxin-secreting beneficial bacteria. Cell Host Microbe.

[21] Žižková E., Kubeš M., Dobrev P.I., Přibyl P., Šimura J., Zahajská L., Záveská Drábková L., Novák O., Motyka V. (2017). Control of cytokinin and auxin homeostasis in cyanobacteria and algae. Ann. Bot..

[22] Cheng X., Li X., Tong M., Wu J., Chan L.L., Cai Z., Zhou J. (2023). Indole-3-acetic acid as a cross-talking molecule in algal-bacterial interactions and a potential driving force in algal bloom formation. Front. Microbiol..

[23] Calatrava V., Hom E.F.Y., Guan Q., Llamas A., Fernández E., Galván A. (2024). Genetic evidence for algal auxin production in *Chlamydomonas* and its role in algal-bacterial mutualism. iScience.

[24] Khasin M., Cahoon R.R., Nickerson K.W., Riekhof W.R. (2018). Molecular machinery of auxin synthesis, secretion, and perception in the unicellular chlorophyte alga *Chlorella sorokiniana* UTEX 1230. PLoS ONE.

[25] Matthews J.L., Khalil A., Siboni N., Bougoure J., Guagliardo P., Kuzhiumparambil U., DeMaere M., Le Reun N.M., Seymour J.R., Suggett D.J. (2023). Coral endosymbiont growth is enhanced by metabolic interactions with bacteria. Nat. Commun..

[26] Khalil A., Bramucci A.R., Focardi A., Le Reun N., Willams N.L.R., Kuzhiumparambil U., Raina J.-B., Seymour J.R. (2024). Widespread production of plant growth-promoting hormones among marine bacteria and their impacts on the growth of a marine diatom. Microbiome.

[27] Sison-Mangus M.P., Kempnich M.W., Appiano M., Mehic S., Yazzie T. (2022). Specific bacterial microbiome enhances the sexual reproduction and auxospore production of the marine diatom, *Odontella*. PLoS ONE.

[28] Patidar S.K. (2025). Metabolic interactions between microalgae and bacteria: Multifunctional ecological interplay and environmental applications. Algal Res..

[29] Segev E., Wyche T.P., Kim K.H., Petersen J., Ellebrandt C., Vlamakis H., Barteneva N., Paulson J.N., Chai L., Clardy J. (2016). Dynamic metabolic exchange governs a marine algal-bacterial interaction. eLife.

[30] Yang Q., Pande G.S.J., Wang Z., Lin B., Rubin R.A., Vora G.J., Defoirdt T. (2017). Indole signalling and (micro)algal auxins decrease the virulence of *Vibrio campbellii*, a major pathogen of aquatic organisms. Environ. Microbiol..

[31] Barak-Gavish N., Dassa B., Kuhlisch C., Nussbaum I., Brandis A., Rosenberg G., Avraham R., Vardi A. (2023). Bacterial lifestyle switch in response to algal metabolites. Elife.

[32] Hogle S.L., Brahamsha B., Barbeau K.A. (2017). Direct heme uptake by phytoplankton-associated *Roseobacter* bacteria. mSystems.

[33] Pukall R., Buntefuss D., Frühling A., Rohde M., Kroppenstedt R.M., Burghardt J., Lebaron P., Bernard L., Stackebrandt E. (1999). *Sulfitobacter mediterraneus* sp. nov., a new sulfite-oxidizing member of the alpha-proteobacteria. Int. J. Syst. Bacteriol..

[34] Qu L., Feng X., Chen Y., Li L., Wang X., Hu Z., Wang H., Luo H. (2021). Metapopulation Structure of Diatom-Associated Marine Bacteria. bioRxiv.

[35] Wang J., Wang Z., Zhao J. (2019). Isolation, Characterization and Implications of the Bacterial Communities Associated with Established Cultures of *Chattonella marina* (Raphidophyceae) and *Skeletonema costatum* (Bacillariophyceae). Acta Oceanol. Sin..

[36] Zhu J., Tang S., Cheng K., Cai Z., Chen G., Zhou J. (2023). Microbial community composition and metabolic potential during a succession of algal blooms from *Skeletonema* sp. to *Phaeocystis* sp.. Front. Microbiol..

[37] Wang H., Zhang S., Pratush A., Ye X., Xie J., Wei H., Sun C., Hu Z. (2018). Acclimation of culturable bacterial communities under the stresses of different organic compounds. Front. Microbiol..

[38] Livak K.J., Schmittgen T.D. (2001). Analysis of relative gene expression data using real-time quantitative PCR and the 2^−ΔΔCT^ method. Methods.

[39] Olin-Sandoval V., Yu J.S.L., Miller-Fleming L., Alam M.T., Kamrad S., Correia-Melo C., Haas R., Segal J., Peña Navarro D.A., Herrera-Dominguez L. (2019). Lysine harvesting is an antioxidant strategy and triggers underground polyamine metabolism. Nature.

[40] Kolmogorov M., Yuan J., Lin Y., Pevzner P.A. (2019). Assembly of long, error-prone reads using repeat graphs. Nat. Biotechnol..

[41] Huerta-Cepas J., Szklarczyk D., Heller D., Hernández-Plaza A., Forslund S.K., Cook H., Mende D.R., Letunic I., Rattei T., Jensen L.J. (2019). eggNOG 5.0: A hierarchical, functionally and phylogenetically annotated orthology resource based on 5090 organisms and 2502 viruses. Nucleic Acids Res..

[42] Kanehisa M. (2000). KEGG: Kyoto Encyclopedia of Genes and Genomes. Nucleic Acids Res..

[43] Mas-Bargues C., Escrivá C., Dromant M., Borrás C., Viña J. (2021). Lipid peroxidation as measured by chromatographic determination of malondialdehyde. Human plasma reference values in health and disease. Arch. Biochem. Biophys..

[44] Imlay J.A. (2015). Diagnosing oxidative stress in bacteria: Not as easy as you might think. Curr. Opin. Microbiol..

[45] Schmacht M., Lorenz E., Senz M. (2017). Microbial production of glutathione. World J. Microbiol. Biotechnol..

[46] Queval G., Thominet D., Vanacker H., Miginiac-Maslow M., Gakière B., Noctor G. (2009). H_2_O_2_-activated up-regulation of glutathione in *Arabidopsis* involves induction of genes encoding enzymes involved in cysteine synthesis in the chloroplast. Mol. Plant.

[47] Krüger A., Vowinckel J., Mülleder M., Grote P., Capuano F., Bluemlein K., Ralser M. (2013). Tpo1-mediated spermine and spermidine export controls cell cycle delay and times antioxidant protein expression during the oxidative stress response. EMBO Rep..

[48] Gutsche K.A., Tran T.B.T., Vogel R.F. (2012). Production of volatile compounds by *Lactobacillus sakei* from branched chain α-keto acids. Food Microbiol..

[49] Bayliak M.M., Lylyk M.P., Vytvytska O.M., Lushchak V.I. (2016). Assessment of antioxidant properties of alpha-keto acids in vitro and in vivo. Eur. Food Res. Technol..

[50] Maqbool A., Horler R.S.P., Muller A., Wilkinson A.J., Wilson K.S., Thomas G.H. (2015). The substrate-binding protein in bacterial ABC transporters: Dissecting roles in the evolution of substrate specificity. Biochem. Soc. Trans..

[51] Ma Y., Li X., Wang F., Zhang L., Zhou S., Che X., Yu D., Liu X., Li Z., Sun H. (2023). Structural and biochemical characterization of the key components of an auxin degradation operon from the rhizosphere bacterium *Variovorax*. PLoS Biol..

