# Supplementary material for: Indole Acetic Acid: A Key Metabolite That Protects Marine Sulfitobacter mediterraneus Against Oxidative Stress
Source: Microorganisms. 2025 Apr 28;13(5):1014. doi: 10.3390/microorganisms13051014 (PMC12114110; doi:10.3390/microorganisms13051014)
Supplement: Supplementary file 1 [file microorganisms-13-01014-s001.zip › microorganisms-3509905-SI.pdf]

Indole acetic acid: A key metabolite that protects marine *Sulfitobacter mediterraneus* against oxidative stress

Yongliang Gan[1], Runlin Cai[1], Guanjing Cai[1], Jude Juventus Aweya[2], Jianmin Xie[1], Ziming Chen[1], Hui Wang[1]*

[1] Guangdong Provincial Key Laboratory of Marine Biotechnology and Biology Department, College of Science, Shantou University, Shantou 515063, China

[2] Fujian Provincial Key Laboratory of Food Microbiology and Enzyme Engineering, College of Ocean Food and Biological Engineering, Jimei University, Xiamen 361021, China

[*] Correspondence author: wanghui@stu.edu.cn

Contents

This material contains **1** Text, **1** Table and **2** Figures.

Text S1 Composition of minimal salt medium

Table S1 Primers used for this study.

Figure S1 Proteomic responses of *S. mediterraneus* SC1-11 under peroxide hydrogen stress before and after treatment with indole acetic acid (IAA).

Figure S2 Supplementation of individual amino acids plays a protective role against oxidative stress in *S. mediterraneus* SC1-11.

Text S1 Composition of minimal salt medium

Minimal salt medium (MSM): 7.01 mM K_2HPO_4, 2.94 mM KH_2PO_4, 0.81 mM $MgSO_4 \cdot 7H_2O$, 0.18 mM $CaCl_2$, 1.71 mM NaCl

Table S1. Primers used for this study.

Primer names	Sequences	Application
16S RNA-F	TTCGGTGACACACCTAACGG	RT-qPCR
16S RNA-R	TTCGGTGACACACCTAACGG	
JNX03_03450F	TTGGCTCGCACACCTACAAT	RT-qPCR
JNX03_03450R	ATCATGTCTGACCCATCCGC	
JNX03_04495F	ATGATGAGGTCAAAGCGGCA	RT-qPCR
JNX03_04495R	ACGTCGCCTTCCTTTTCGAT	
JNX03_07335F	ACAAGAAACTGACCGCCGAT	RT-qPCR
JNX03_07335R	CGCCATCAACCAGCGTATTG	
JNX03_07340F	ACCCTGTGCAGTTGCTATCC	RT-qPCR
JNX03_07340R	CACCAGATAACGGACCTCGG	
JNX03_12660F	AGGTATTGAACGGCATGGCA	RT-qPCR
JNX03_12660R	CCCCTGGACAGAAACCCTTC	
JNX03_14925F	TTGACGGGTTCCTCAAAGCA	RT-qPCR
JNX03_14925R	TCTGGTTCATCGGCCAGTTC	
JNX03_15035F	TTTCGTCGCAGAAACCGAGA	RT-qPCR
JNX03_15035R	CAGTCCATAGCGGGTGTTGT	
JNX03_17115F	GGTGTGATGACCAGACCGTT	RT-qPCR
JNX03_17115R	GCGAAATCTCCGACCAGTGA	
JNX03_17255F	CCGTCGATGACCGATCACAT	RT-qPCR
JNX03_17255R	GCTGAAAATGCTCGCCTACG	
JNX03_18460F	GAGGCGATCCGAAAACTGGA	RT-qPCR
JNX03_18460R	CTGATCTTCATCCGACCCCG	

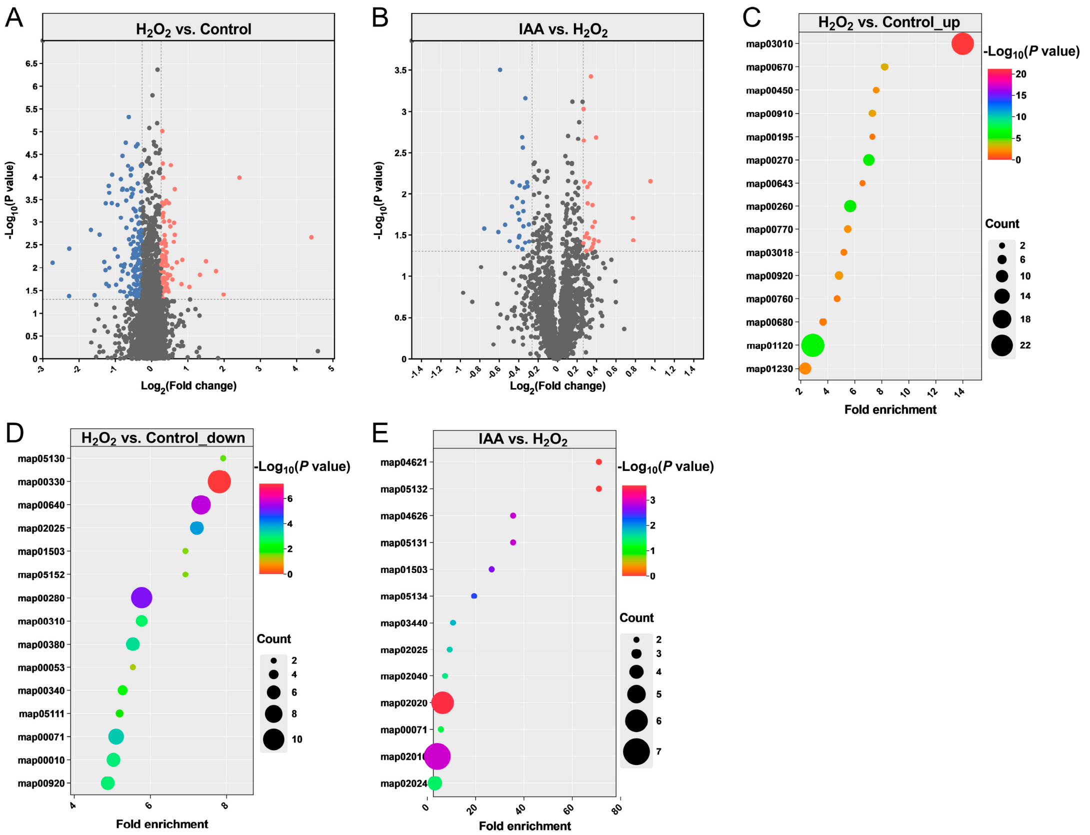

Figure S1 Proteomic response of *S. mediterraneus* SC1-11 under peroxide hydrogen stress before and after treatment with indole acetic acid (IAA). (**A**) Volcano plot shown the comparison between cultures treated with H_2O_2 and the control. (**B**) Volcano plot shown the comparison between cultures treated with H_2O_2 plus IAA and the the culture treated with H_2O_2 only. (**C-D**) KEGG enrichment of the upregulated (**C**) or downregulated (**D**) proteins in response to oxidative stress. (**E**) KEGG enrichment of differentially expressed proteins in response to IAA addition.

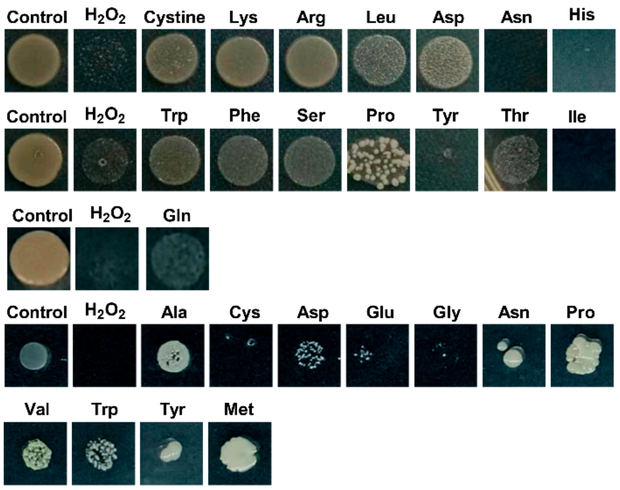

Figure S2 Supplementation of individual amino acids plays a protective role against oxidative stress in *S. mediterraneus* SC1-11. Abbreviations for amino acids: Ala, alanine; Cys, cysteine; Asp, aspartic acid; Glu, glumatic acid; Phe, phenyalnine; Gly, glycine; His, histidine; Leu, leucine; Ile, isoleucine; Lys, lysine; Met, methionine; Asn, asparagine; Pro, proline; Gln, glutamine; Arg, arginine; Ser, serine; Thr, threonine; Val, valine; Trp, tryptophan; Tyr, tyrosine.
